# Supplementary figures and images for: Brain transcriptome analysis reveals subtle effects on mitochondrial function and iron homeostasis of mutations in the SORL1 gene implicated in early onset familial Alzheimer’s disease
Source: Mol Brain. 2020 Oct 19;13:142. doi: 10.1186/s13041-020-00681-7 (PMC7570131; doi:10.1186/s13041-020-00681-7)

**A***sorl1* exon 2 region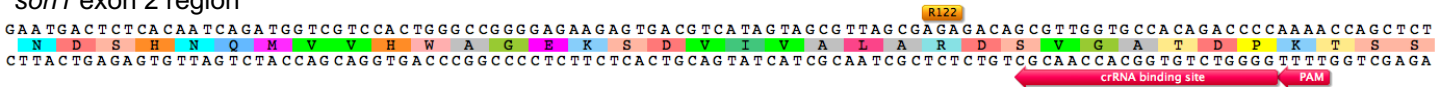**B***sorl1* exon 32 region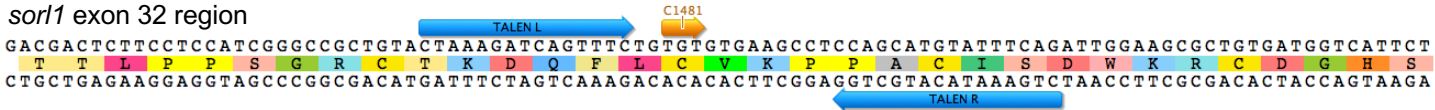

Supplement: Supplementary file 1 — Additional file 1: Genome editing of zebrafish sorl1. a and b show sections of the sorl1 genomic sequence (ENSG00000137642) with the sense sequence (upper), translation (middle) and anti-sense sequence (lower). a sorl1 exon 2, with the crRNA binding site and PAM sequence for cleavage by Cpf1 indicated by pink arrows, and the R122 site indicated by the orange bar. b sorl1 exon 32, with the C1481 codon indicated by the orange arrow and the TALEN binding sites by blue arrows. [file 13041_2020_681_MOESM1_ESM.pdf]

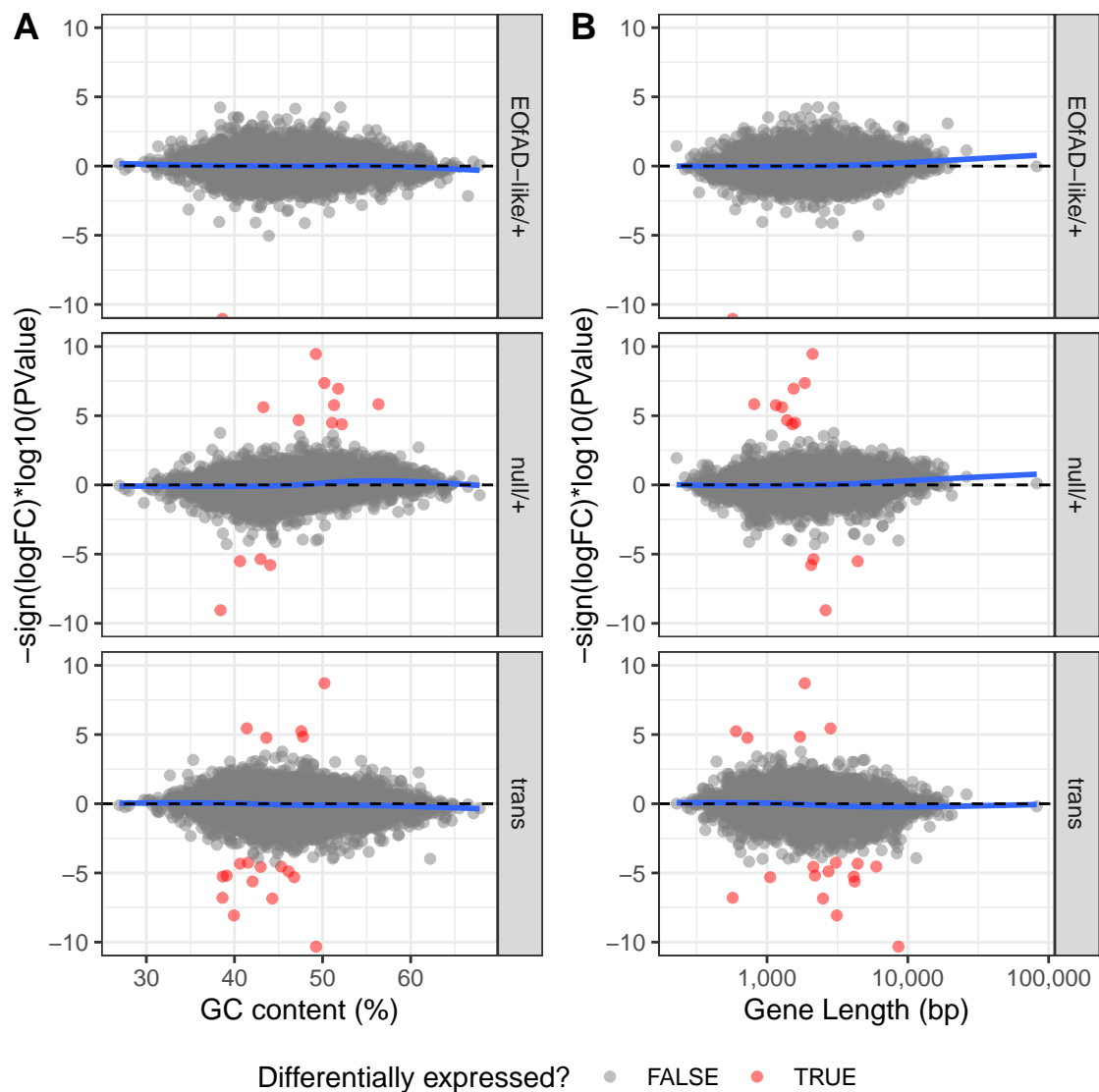

Supplement: Supplementary file 3 — Additional file 3: No observed bias for differential expression with GC content or length. Plots showing a ranking metric using the sign of logFC multiplied by − log10 of the p-value against a the GC content of the gene and b the length of the gene. The blue curve indicates the line of best fit from a generalised additive model (gam), whilst the black dashed line represents the y = 0 line. Given the gam fit is a nearly horizontal line mostly overlapping y = 0, a significant bias for GC content or length is likely not present in this dataset. The ranking statistic limits were constrained to − 10 and 10 for visualisation purposes, and in b, gene length was plotted on the log10 scale. [file 13041_2020_681_MOESM3_ESM.pdf]

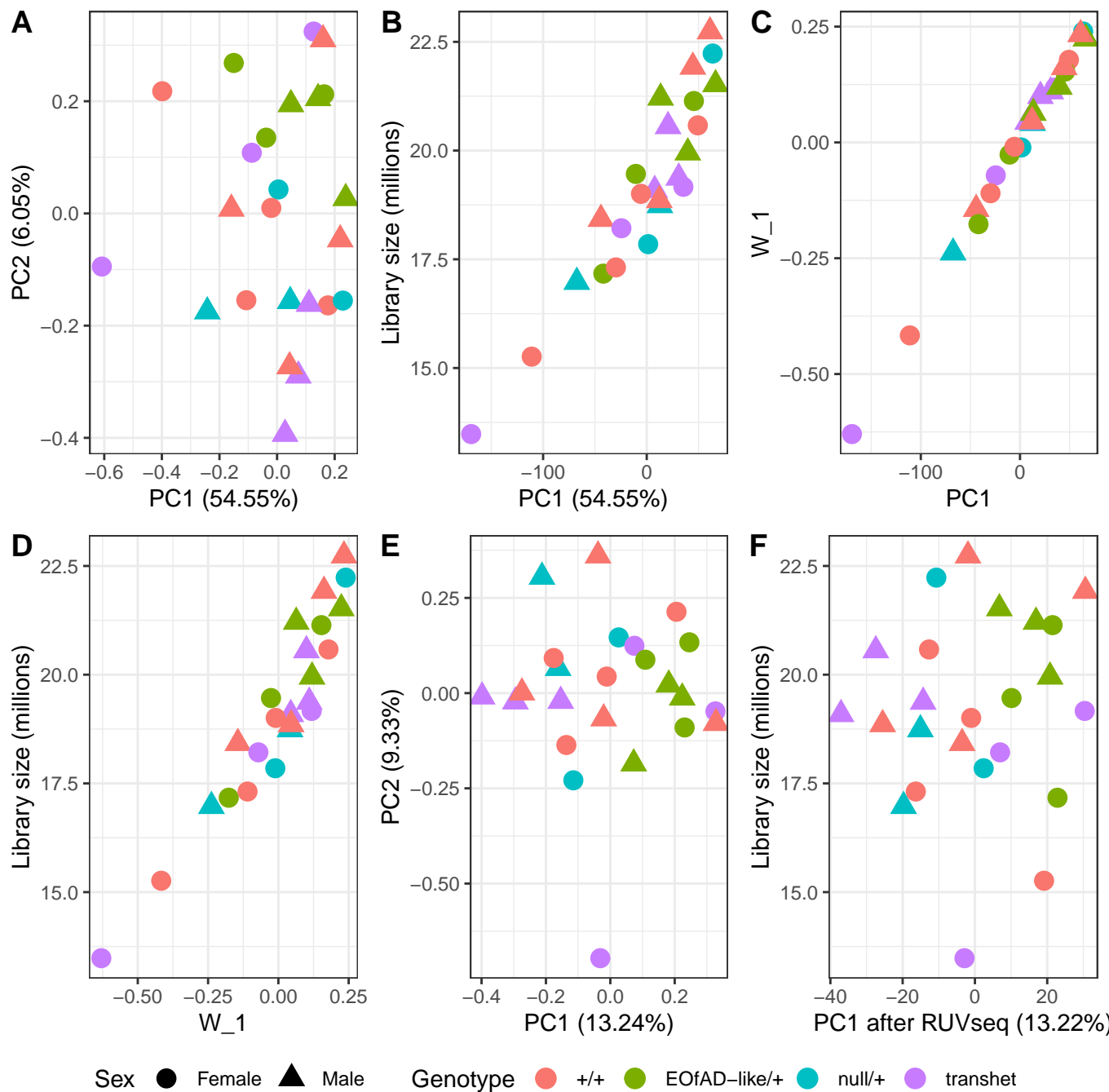

Supplement: Supplementary file 4 — Additional file 4: Principal component analysis. a Plot of principal component 1 (PC1) against PC2 from a principle component analysis (PCA) of the logCPMs from each sample. b PC1 against the library size per sample. The linear relationship observed between PC1 and library size suggests that the largest source of variability in this dataset is due to library size. c PC1 against the W_1 covariate from RUVseq. A linear relationship between PC1 and W_1 is observed. d W_1 against library size. e PC1 against PC2 after removal of 1 factor of unwanted variation using RUVSeq [38]. f PC1 no longer depends on library size after RUVSeq transformation. [file 13041_2020_681_MOESM4_ESM.pdf]

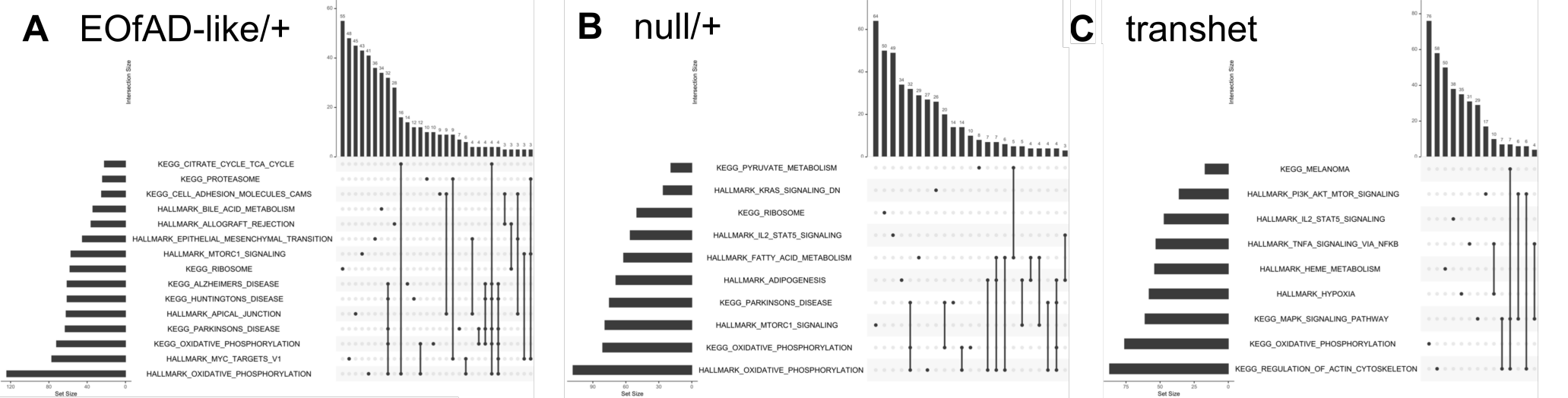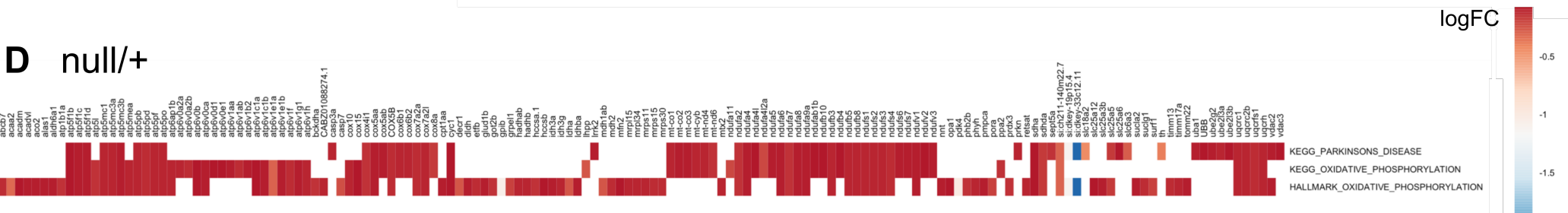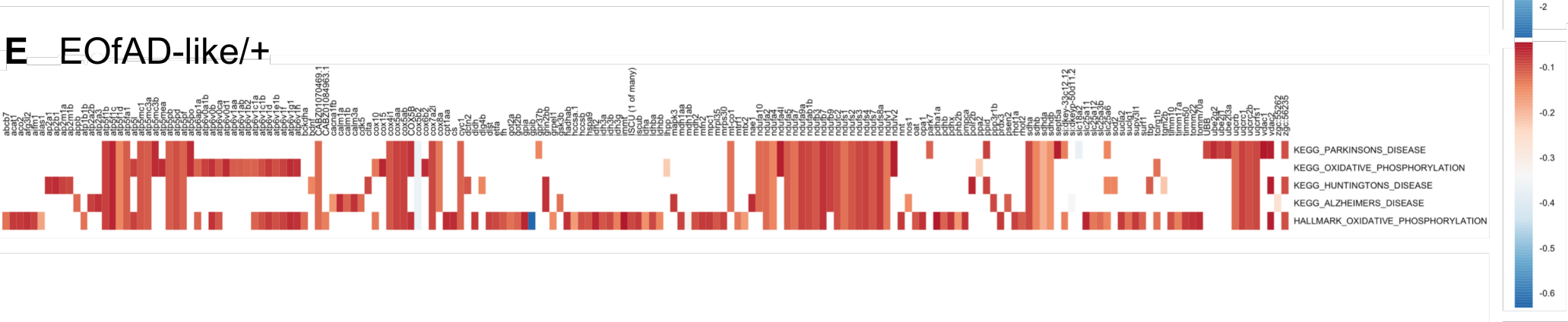

Supplement: Supplementary file 7 — Additional file 7: Results from enrichment analysis are mostly not driven by expression of the same genes. The upset plots show the overlap of the “leading edge” genes from the fgsea algorithm in each of the significant gene sets in a EOfAD-like/+, b null/+, and c transheterozygous mutant brains. Intersections are shown when the leading edge of the gene sets share three or more genes. Overall, the leading edge genes of the significantly altered gene sets are relatively independent of one another. However, genes in the oxidative phosphorylation gene sets and the gene sets for neurodegenerative diseases (Alzheimer’s, Parkinson’s and Huntington’s diseases) all contain genes encoding components of the electron transport chain and are capturing a portion of the same gene expression signal, which is shown in d and e. Missing positions in d and e indicate that the gene was not in the leading edge for that gene set. [file 13041_2020_681_MOESM7_ESM.pdf]

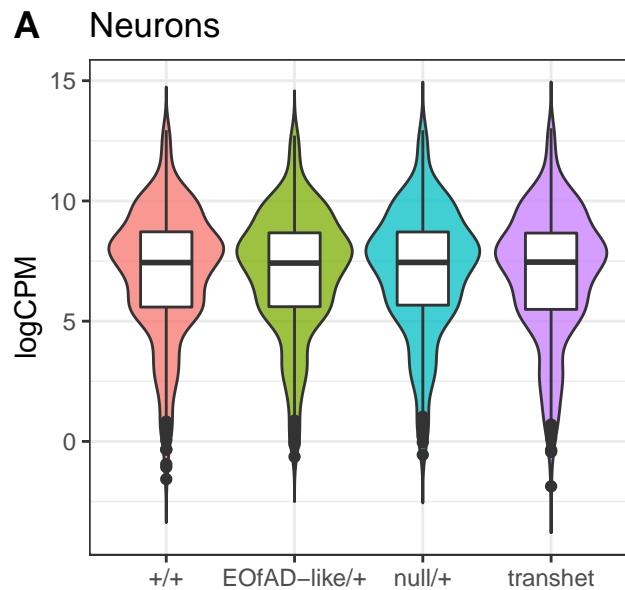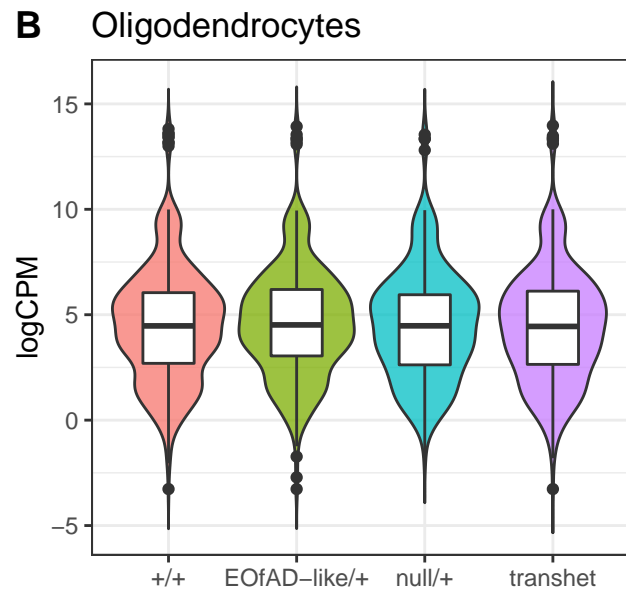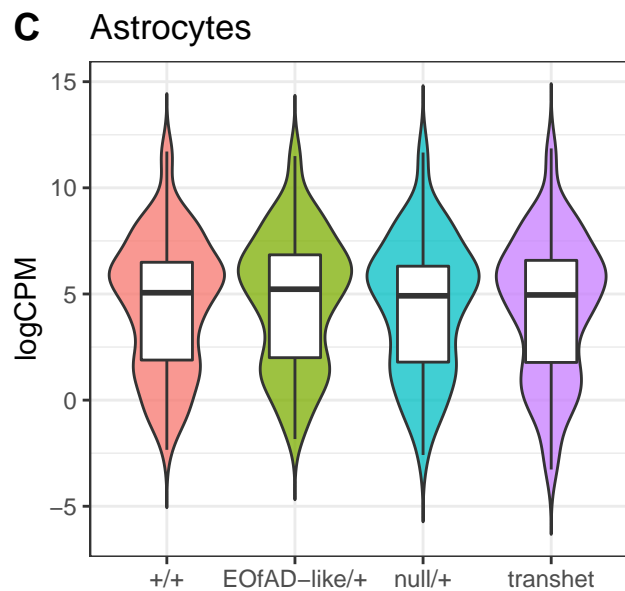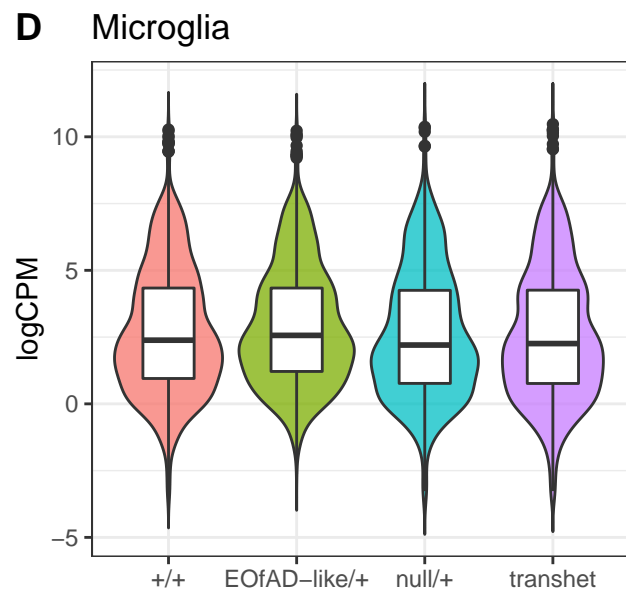

Supplement: Supplementary file 8 — Additional file 8: Changes to gene expression in young-adult zebrafish brain transcriptomes are likely not due to altered cell type proportions. We obtained representative expression markers of neurons (72 genes), oligodendrocytes (100 genes) and astrocytes (44 genes) from [53] and representative expression markers of microglia (533 genes) from [54]. The logCPM distributions for these marker genes in each of the samples are similar, supporting that, broadly, the distribution of these cell types is consistent between samples. Data are shown as violin plots, displaying the kernel probability density of the logCPMs, overlaid with boxplots, showing summary statistics, and coloured by genotype. [file 13041_2020_681_MOESM8_ESM.pdf]

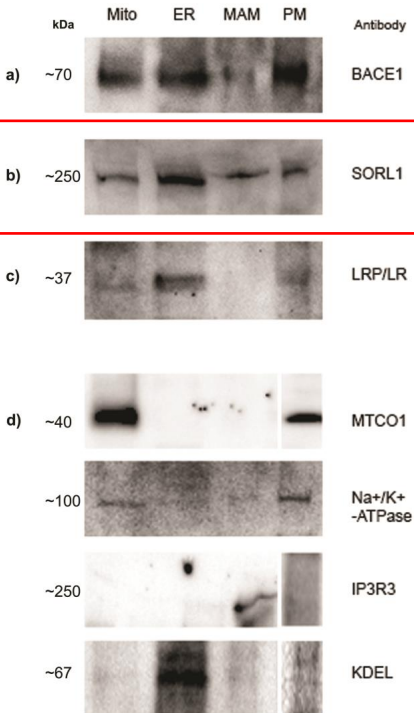

Supplement: Supplementary file 10 — Additional file 10: SORL1 is localized in the MAM in mouse brains. Western blot analysis of subcellular fractions of mouse brain cellular membranes. Each subcellular fraction probed with antibodies against a BACE1, b SORL1 and c LRP/LR. d Shows the identity of fractions western immunoblotting using antibodies recognising proteins MTCO1 in mitochondria (Mito), Na+/K+-ATPase in the plasma membrane (PM), IP3R3 in mitochondrial-associated membranes (MAM), and KDEL, predominantly in non-MAM endoplasmic reticulum (ER). Some cross contamination was observed as MTOC1 was detected in both the mitochondria and plasma membrane, and Na+/K+-ATPase in both the mitochondria and plasma membrane. Lim, A. H. L. (2015). Analysis of the subcellular localization of proteins implicated in Alzheimer's Disease. Genetics and Evolution, University of Adelaide. Doctor of Philosophy (PhD): 235. This figure is reproduced from the Ph.D. thesis of Anne Lim for ease of access. [file 13041_2020_681_MOESM10_ESM.pdf]
