# Supplementary material for: Brain transcriptome analysis reveals subtle effects on mitochondrial function and iron homeostasis of mutations in the SORL1 gene implicated in early onset familial Alzheimer’s disease
Source: Mol Brain. 2020 Oct 19;13:142. doi: 10.1186/s13041-020-00681-7 (PMC7570131; doi:10.1186/s13041-020-00681-7)
Supplement: Supplementary file 9 — Additional file 9: Pathview visualisation of the KEGG oxidative phosphorylation gene set. The logFC of the genes in the KEGG oxidative phosphorylation gene set are shown in each sorl1 genotype comparison with wild type. Intensity of the colours indicates the magnitude of the logFC, and white indicates that a gene was not detected as expressed in the RNA-seq experiment. Plot was adapted from pathview [83]. [file 13041_2020_681_MOESM9_ESM.pdf]

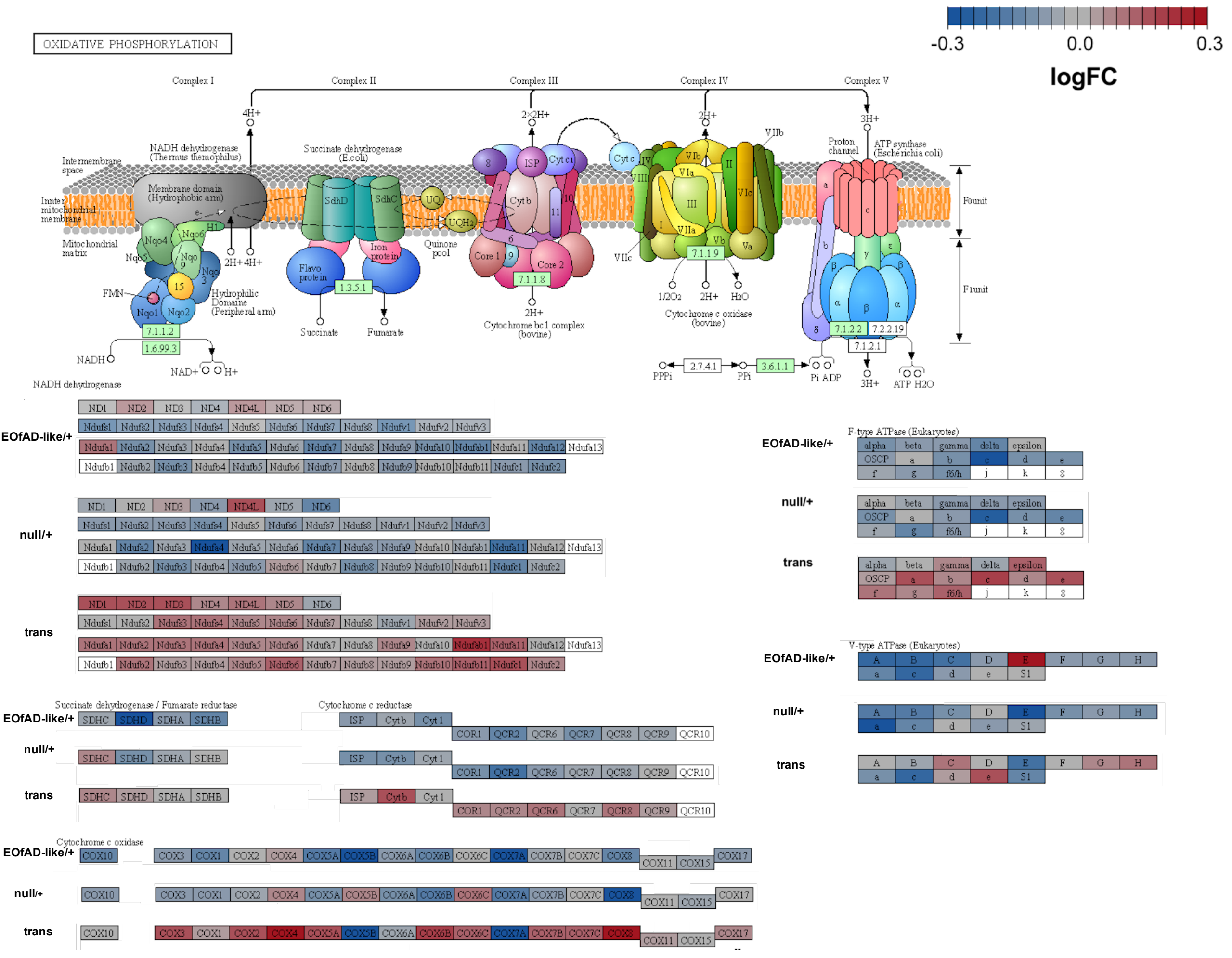
OXIDATIVE PHOSPHORYLATION
-0.3
0.0
0.3
logFC
Complex I
Complex II
Complex III
Complex IV
Complex V
4H+
2×2H+
2H+
3H+
NADH dehydrogenase (Thermus themophilus)
Succinate dehydrogenase (E.coli)
Intermembrane space
Inner mitochondrial membrane
Mitochondrial matrix
Membrane domain (Hydrophobic arm)
Nqo4
Nqo6
Nqo5
Nqo9
Nqo3
Nqo1
Nqo2
H1
15
FMN
Hydrophilic Domaine (Peripheral arm)
2H+ 4H+
7.1.1.2
1.6.99.3
NADH
NAD+
H+
SdhD
SdhC
UQ
UQH2
Flavo protein
Iron protein
1.3.5.1
Succinate
Fumarate
Quinone pool
8
ISP
Cyt c1
Cyt b
Core 1
Core 2
7.1.1.8
2H+
Cytochrome bc1 complex (bovine)
Cyt c
VIIb
VIIc
VIII
Vb
Va
VIa
VIIa
III
II
VIc
7.1.1.9
1/2O2
2H+
H2O
Cytochrome c oxidase (bovine)
Proton channel
ATP synthase (Escherichia coli)
Founit
F1unit
7.1.2.2
7.2.2.19
7.1.2.1
2.7.4.1
3.6.1.1
PPPi
PPi
Pi ADP
3H+
ATP H2O
NADH dehydrogenase
EOfAD-like/+
null/+
trans
ND1 ND2 ND3 ND4 ND4L ND5 ND6
Ndufs1 Ndufs2 Ndufs3 Ndufs4 Ndufs5 Ndufs6 Ndufs7 Ndufs8 Ndufv1 Ndufv2 Ndufv3
Ndufa1 Ndufa2 Ndufa3 Ndufa4 Ndufa5 Ndufa6 Ndufa7 Ndufa8 Ndufa9 Ndufa10 Ndufab1 Ndufa11 Ndufa12 Ndufa13
Ndufb1 Ndufb2 Ndufb3 Ndufb4 Ndufb5 Ndufb6 Ndufb7 Ndufb8 Ndufb9 Ndufb10 Ndufb11 Ndufc1 Ndufc2
F-type ATPase (Eukaryotes)
alpha beta gamma delta epsilon
OSCP a b c d e
f g f6/h j k 8
V-type ATPase (Eukaryotes)
A B C D E F G H
a c d e S1
Succinate dehydrogenase / Fumarate reductase
SDHC SDHD SDHA SDHB
Cytochrome c reductase
ISP Cyt b Cyt 1
COR1 QCR2 QCR6 QCR7 QCR8 QCR9 QCR10
Cytochrome c oxidase
COX10
COX3 COX1 COX2 COX4 COX5A COX5B COX6A COX6B COX6C COX7A COX7B COX7C COX8
COX11 COX15
COX17
